# Shortest triplet clustering: reconstructing large phylogenies using representative sets

**DOI:** 10.1186/1471-2105-6-92

**Published:** 2005-04-08

**Authors:** Le Sy Vinh, Arndt von Haeseler

**Affiliations:** 1 Heinrich-Heine-Universität Düsseldorf, WE Informatik, Universitätstr. 1, D-040225 Düsseldorf, Germany; 2 Forschungszentrum Jülich, Germany

## Abstract

**Background:**

Understanding the evolutionary relationships among species based on their genetic information is one of the primary objectives in phylogenetic analysis. Reconstructing phylogenies for large data sets is still a challenging task in Bioinformatics.

**Results:**

We propose a new distance-based clustering method, *the shortest triplet clustering algorithm (STC)*, to reconstruct phylogenies. The main idea is the introduction of a natural definition of so-called *k-representative sets*. Based on *k*-representative sets, *shortest triplets *are reconstructed and serve as building blocks for the STC algorithm to agglomerate sequences for tree reconstruction in *O*(*n*^2^) time for *n *sequences.

Simulations show that STC gives better topological accuracy than other tested methods that also build a first starting tree. STC appears as a very good method to start the tree reconstruction. However, all tested methods give similar results if balanced nearest neighbor interchange (BNNI) is applied as a post-processing step. BNNI leads to an improvement in all instances. The program is available at .

**Conclusion:**

The results demonstrate that the new approach efficiently reconstructs phylogenies for large data sets. We found that BNNI boosts the topological accuracy of all methods including STC, therefore, one should use BNNI as a post-processing step to get better topological accuracy.

## Background

Reconstructing the evolutionary relationships among species based on their genetic information is one of the primary objectives in phylogenetic analysis. In recent years, numerous heuristics to reconstruct phylogenies for large data sets have been proposed [1-11]. In addition, parallel tree-reconstruction programs have been implemented [12-15].

To date, distance-based methods introduced by Cavalli-Sforza and Edwards [16] and by Fitch and Margoliash [17] appear most appropriate to reconstruct phylogenies based on thousands of sequences. These methods are a compromise between computational speed and topological accuracy [1,3,5-7] and run typically in *O*(*n*^3^) time for *n *sequences [1,3,5] or in *O*(*n*^2^) for recently suggested approaches [6,7]. Clustering algorithms form a major class of distance-based methods [18]. They do not have an explicit objective function that needs to be optimized. They rather group sequences (or taxa) iteratively to reconstruct a distance-based phylogenetic tree. UPGMA is a popular method to infer phylogenies with the constraint that a molecular clock is imposed on the evolutionary process. Other clustering approaches have been proposed to relax the molecular clock assumption [1,3,5,19-21].

An attempt to boost the accuracy and to reduce the computational burden is the introduction of *k*-representative set concepts [10,11]. *k-*representative sets consist of at most *k *elements but retain the most important information from whole sets. In this paper, we extend our original approach [10] by introducing a more natural *k-*representative set concept. In a nutshell, representative sets are regarded as components to construct shortest triplets, each of which comprises three closely related sequences from three *k*-representative sets. The collection of shortest triplets serves as building block for a new distance-based clustering method called shortest triplet clustering algorithm (STC).

## Results

Simulations were run on a PC cluster with 16 nodes. Each node has two 1.8 GHz processors and 2 GB RAM. Seq-Gen [22] was used to evolve sequences along trees using the Kimura two-parameter model [23] with a transition/transversion ratio of 2.0. We generated 100 simulated data sets of 500 sequences each with sequence lengths 500, 1000 and 2000 nucleotides (nt), respectively. As one model tree, we used the rbcl gene tree with diameter 0.36 substitutions per site as inferred from an alignment of 500 rbcl-genes [10]. We call this *the rbcl-simulation*.

In a second experiment, the so-called *large simulation*, tree topologies were drawn from the Yule-Harding distribution [24], and edge lengths were drawn from an exponential distribution and subsequently rescaled such that the mean diameter of the tree was either 0.1, 0.5, 1.0, or 1.5. For each value of the diameter we generated 100 trees with 1000 sequences and 100 trees with 5000 sequences. Thus, a total of 800 trees were used.

Finally, we tested the accuracy and runtime of the STC and compared it with six other commonly used distance-based methods. More specifically, we investigate the performance of the Neighbor-Joining method (NJ) [1] implemented in PAUP* 4.0 [25], BIONJ [3], Weighbor 1.2 [5], Harmony Greedy Triplet and Four Point Condition (HGT/FP) [7] as well as Greedy Minimum Evolution (GME) and Balanced Minimum Evolution (BME) [6]. Unfortunately, no distance-based program is available for the disc-covering method [4]. All methods were combined with DNADIST version 3.5 [26] and pairwise distances were corrected for multiple hits according to the model used in the simulation. Moreover, we examined the performance of all methods when the balanced nearest neighbor interchange (BNNI) [6] is used as a post-processing step.

Further, to illustrate the performance of STC we re-analyzed the 96-taxon alignments of sequence length 500 nt, that were analyzed in [6] and available at . The 6000 trees were split into three groups called "slow" (0.2 substitutions per site), "moderate" (0.4 substitutions per site) and "fast" (1.0 substitutions per site). We call this *the re-analyzed simulation*.

The accuracy of a tree reconstruction method for a simulated data set is measured by the Robinson and Foulds (*RF*) distance [27] between the inferred tree and the model tree used to generate the data set. The RF distance between two trees is the number of bi-partitions present in one of the two trees but not the other, divided by the number of possible bi-partitions. Thus, the smaller the RF distance between two trees the closer are their topologies. In other words, the smaller the RF distance is between the inferred tree and the model tree the higher is the topological accuracy of the tree reconstruction method.

In the following we discuss the results of *the rbcl-simulation*, and *the large simulation *and *the re-analyzed simulation*.

### rbcl-simulation

Table 1 shows that the STC outperforms all other methods analyzed in terms of topologlcal accuracy. For instance, the RF distance between the STC-tree and the model tree is on average 0.177 (with respect to the sequence length of 500 nt) and better than NJ (0.190), slightly better than the second best method BME (0.184) and much better than HGT/FP (0.512). Table 1 also demonstrates that all tested methods including STC give higher topologlcal accuracy when the sequence length is increased. However, Table 2 shows that other methods in combination with BNNI outperform STC without BNNI. The combination of STC and BNNI shows similar performance as the combinations of NJ (BIONJ, Weighbor) and BNNI and, slightly better results than the combination of GME (HGT/FP) and BNNI.

### Large simulation

Due to the increase in runtime, Weighbor could not be tested. Table 3 and 4 show that STC gives better results than the other methods independent of the diameter. All methods display a decrease in accuracy when the number of sequences changes from 1000 to 5000. As shown in Table 5 and 6, BNNI boosts the accuracy of all methods including STC. All methods give similar results when being used together with BNNI.

### Re-analyzed simulation

Except for STC, the accuracies for the other methods displayed in Table 7 and 8 were taken from [6]. Table 7 shows that STC outperforms the other methods in terms of topological accuracy with the exception that Weighbor is slightly better than STC with respect to the slow simulation group. If BNNI is applied, all methods exhibit an almost identical performance (see Table 8).

### Another look at the performance

**Figure 1:**
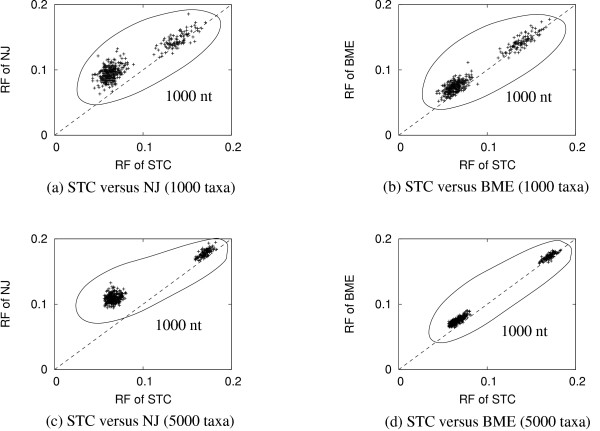
The comparisons of topological accuracy between STC, NJ and BME for the *large simulation*. Each point in the graph presents the Robinson and Foulds (RF) distance for a simulated data set. Points above the dotted line are examples where the RF distance of the STC-tree is less than the RF distance of the NJ-tree or BME-tree. Thus, the STC gives higher topological accuracy than NJ or BME with respect to the simulated data set.

Instead of looking at the average RF distance, we suggest to take a closer look at the simulated data. For each simulated data set, that is subjected to the STC and six other tree reconstruction methods mentioned above, we compute the RF distance between the reconstructed tree and the model tree for all methods. Figure 1 illustrates the results for the large simulation when comparing STC with NJ (left column) and STC with the second best method BME (right column). In each diagram specified by the number of taxa and reconstruction methods, 400 points are displayed, that resulted from 100 simulations for each of the tree-diameters (0.1, 0.5, 1.0 and 1.5). Although four tree-diameters were studied only two clouds of points are discernible, where the cloud in the north-east corner of each diagram represents the simulations with the tree-diameter 0.1. The remaining 300 points gather in the south-west cloud because the RF-distances from trees with diameter 0.5, 1.0, 1.5 are not substantially different from each other (see Table 3 and 4). More precisely, the horizontal and vertical axes indicate the RF distances of STC and NJ (or BME), respectively. Each point in the graph presents the RF distance for a simulated data set. Points above the dotted line are examples where the RF distance of the STC-tree is less than the RF distance of the NJ-tree or BME-tree. Thus, the STC gives higher topological accuracy than NJ or BME with respect to the simulated data set. For example, Figure 1a illustrates the comparison between STC and NJ with respect to 1000 taxa data sets. 379 out of 400 points are above the diagonal, thus, STC gives better results than NJ in about 95% of the simulations. For the remaining 21 alignments (points), two methods showed the same RF distance. Finally, we found 19 points below the diagonal in which case NJ outperforms STC. For the *large simulation *(5000 taxa), NJ is worse than STC in all cases. However, the second best method BME is better than STC in 11% and 5% of the cases with respect to 1000 and 5000 sequence data sets.

**Figure 2:**
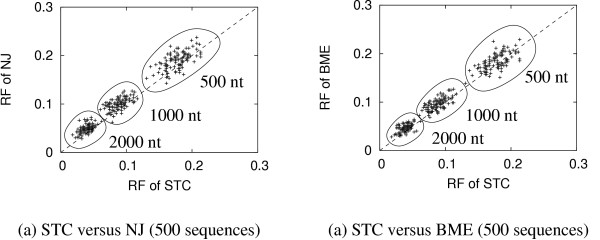
The comparisons of topological accuracy between STC, NJ and BME for the *rbcl simulation*. Each point in the graph presents the Robinson and Foulds (RF) distance for a simulated data set. Points above the dotted line are examples where the RF distance of the STC-tree is less than the RF distance of the NJ-tree or BME-tree. Thus, the STC gives higher topological accuracy than NJ or BME with respect to the simulated data set.

Figure 2 shows the same analysis for the rbcl simulation. It shows that with increasing sequence length the cloud of points moves towards zero. From Figure 2 we learn that in some instances NJ (or BME) performs better (with regard to the RF distance) than STC, i.e. 20%, 12%, 8% (or 34%, 17%, 14%) of the simulations for sequence lengths 500, 1000 and 2000 nt, respectively.

Similar results hold for the other methods. These results are summarized in Table 9 where we show the percentage of simulations in which STC is at least as good as the other methods.

Again, if BNNI is applied we observe that no substantial difference among the various approaches. The accuracy of the methods is mostly determined by BNNI (see Table 10).

## Conclusion

We are presenting *k*-representative sets which allow us to design a fast and accurate method to reconstruct phylogenies from large data sets with 1000 or more taxa. Simulations show that STC gives better results than other tested methods in terms of topological accuracy. However, if BNNI is introduced as a subsequent optimization step, the differences in the performance disappear. All methods show more or less the same accuracy. Thus, one should apply BNNI to improve the topological accuracy.

The time to reconstruct a tree of up to 1000 sequences is not really an issue for all tested distance-based methods, with the exception of Weighbor. Weighbor needed about 19 minutes to reconstruct a tree with 500 sequences, thus it is only applicable to data sets with up to some hundred sequences. For data sets with up to 1000 sequences, the remaining methods needed less than one minute to output a tree, thus the difference between methods in terms of runtime is not significant. For data sets with 5000 sequences, STC (GME, HGT/FP or BME) with BNNI took about 2.0 (2.5, 3.0 or 3.5) minutes to reconstruct a tree. NJ (BIONJ) with BNNI were slower and consumed approximately six minutes to output a tree. In short, the combination of STC and BNNI efficiently reconstruct trees for large data sets in both terms of topological accuracy and runtime.

**Figure 3:**
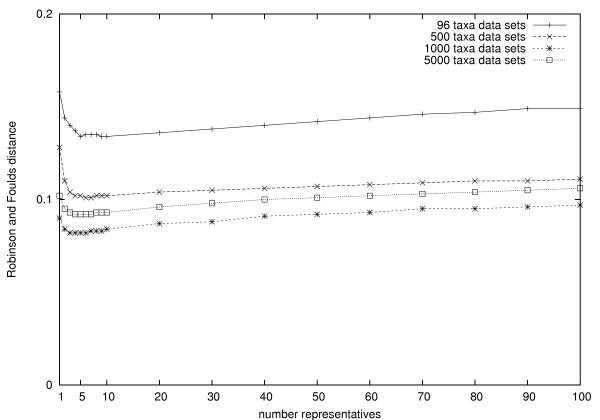
The impact of the number of representatives *k*. The RF distance of STC decreases when *k *grows from 1 to 5. When *k *ranges from 5 to 10, the RF distance remains more or less unchanged. For *k *≥ 10, the RF distance increases steadily indicating a loss of accuracy.

Finally, we did not systematically evaluate the impact of the number of representatives *k*. We present some preliminary results for *k = *1, 2, 3, 4, 5, 6, 7, 8, 9, 10, 20, 30, 40, 50, 60, 70, 80, 90 and 100. Figure 3 shows that the RF distance of STC decreases when *k *grows from 1 to 5. This proves our intuition that a too small number of triplets leads to an inaccurate estimate of path lengths and edge lengths. When *k *ranges from 5 to 10, the RF distance remains more or less unchanged. For *k *≥ 10, the RF distance increases steadily indicating a loss of accuracy. The decrease in accuracy is explained by the inclusion of triplets with large distances which include noise and disturb the reconstruction. Thus, we chose *k *= 5 as a good compromise between the accuracy and computational complexity for all data sets. That is, the practical complexity of the STC algorithm is only *O*(*n*^2^).

## Methods

In this section we introduce a new clustering algorithm to reconstruct phylogenies based on distance matrices.

### Additive distances

Let *S *= {*s*_1_, *s*_2_,..., *s*_*n*_} be a set of *n *objects (typically contemporary sequences/taxa), let *D *= *D*(*uv*) be a distance matrix where *D*(*uv*) is the distance between two objects *u *and *v*.

#### Definition 1

*The distance matrix D is additive if and only if it satisfies the four-point condition *[28]: *for any quartet *{*u*, *v*, *w*, *x*},

*D*(*uv*) + *D*(*wx*) ≤ *max*{*D*(*uw*) + *D*(*vx*), *D*(*ux*) + *D*(*vw*)}.

In this case, the objects *s *∈ *S *are related by a tree *T *= (*V*, *E*) where *V *is the set of vertices such that *S *⊂ *V *and *E *= {{*v*_1_, *v*_2_}|*v*_1_, *v*_2 _∈ *V*} is the set of edges. A vertex with one adjacent edge is called a *leaf*, all other vertices are called *internal nodes*. We let *L *⊂ *V *be the leaf set of the tree *T*. Note that we typically require *L *⊆ *S *in the phylogenetic setting.

If *D *is additive, then there exists a map  and a length function  such that



for all *u*, *v *∈ *S *where *p*(*φ *(*u*), *φ*(*v*)) is the unique path connecting *φ*(*u*) and *φ*(*v*) in *T *and  denotes the distances between vertices in *T *(cf. [29]). ℓ(*e*) is called edge length of the edge *e*. To avoid unnecessary complication, we consider only one-to-one maps from *S *on the leaf set *L *of *T*. If *D *is additive, the reconstruction of tree *T *and ℓ is trivial. If *D *is not additive, methods are available that try to fit a tree *T *to *D *with respect to an objective function (cf. [30]). Thus, in the following we consider arbitrary distance matrices and we want to reconstruct a tree  together with a length function .

### Estimating edge lengths using triplets

We consider a subset *X *of *S*, then  induces a map on a subtree of *T *such that the relationships of objects in *X *are displayed by the subtree with leaf set *φ*(*X*). The complement *S*_0_(*X*) = *S *- *X *we will call the *unclassified object set*, because the relationships of objects in *S*_0_(*X*) to *X *is not known from the subtree. Note that we will use *S*_0 _instead of *S*_0_(*X*) if *X *is clear from the context.

Let denote *T*_*r *_= (*V*_*r*_, *E*_*r*_) a rooted tree with root *r *and leaf set *L*_*r*_, and let *S*_*r *_be a subset of *S *such that *φ*(*S*_*r*_) = *L*_*r*_. For convenience, we use *S*_*r *_and *L*_*r *_interchangeably.

**Figure 4:**
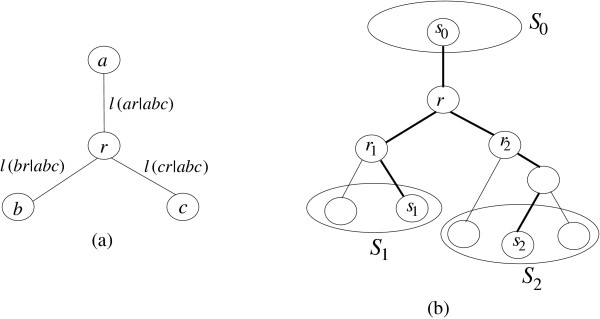
On the left, estimation of edge lengths ℓ(*ar*|*abc*), ℓ(*br*|*abc*) and ℓ(*cr*|*abc*) of the triplet tree {*a*, *b*, *c*}. On the right, estimation of path length ℓ(*s*_0_*r*|*s*_0_*s*_1_*s*_2_) and edge lengths ℓ(*r*_1_*r*|*s*_0_*s*_1_*s*_2_), ℓ(*r*_2_*r*|*s*_0_*s*_1_*s*_2_) based on the triplet tree {*s*_0_, *s*_1_, *s*_2_}.

Now, we consider the most simple edge length estimation problem. That is, we would like to estimate the edge lengths for a triplet tree {*a*, *b*, *c*} with distance matrix *D *(see Figure 4a). Edge lengths are estimated as follows







Now consider a rooted *T*_*r *_with the inferred tree-like metric . The rooted tree *T*_*r *_consists of two rooted subtrees  and  (see Figure 4b). For convenience, we will use *T*_*i *_instead of 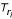 if *r*_*i *_is clear from the context. The leaf set *S*_*r *_= {*S*_1 _∪ *S*_2_} where *S*_*r *_⊂ *S *and *S*_0 _= *S *- *S*_*r *_is not represented in *T*_*r*_. Then we can compute







for each triplet (*s*_0_, *s*_1_, *s*_2_) ∈ (*S*_0 _× *S*_1 _× *S*_2_).

With (*s*_1_*r*_1_) and (*s*_2_*r*_2_) we denote the known distances of *s*_1 _and *s*_2 _to their roots *r*_1 _and *r*_2_. Thus, we can compute for each triplet {*s*_0_, *s*_1_, *s*_2_} the lengths ℓ(*r*_1_*r*) and ℓ(*r*_2_*r*) as





Note that, if *D *is additive and *T*_1_, *T*_2 _are isometric subtrees of *T*, the lengths ℓ(*r*_1_*r*) and ℓ(*r*_2_*r*) do not depend on the choice of the triplet {*s*_0_, *s*_1_, *s*_2_}.

Regardless of additivity considerations, we may define the average length for a fixed *s*_0 _∈ *S*_0 _as



We can estimate edge lengths ℓ(*r*_1_*r*) and ℓ(*r*_2_*r*) by using all possible triplets as





### Recovering a tree from a distance matrix

#### The largest path length criterion

We want to reconstruct a tree *T = *(*V, E*) with respect to a distance matrix *D *such that *D*_*T *_represents *D*. To this end, we use triplets and the notation of a rooted tree *T*_*r *_together with Equations 4 and 5.

**Figure 5:**
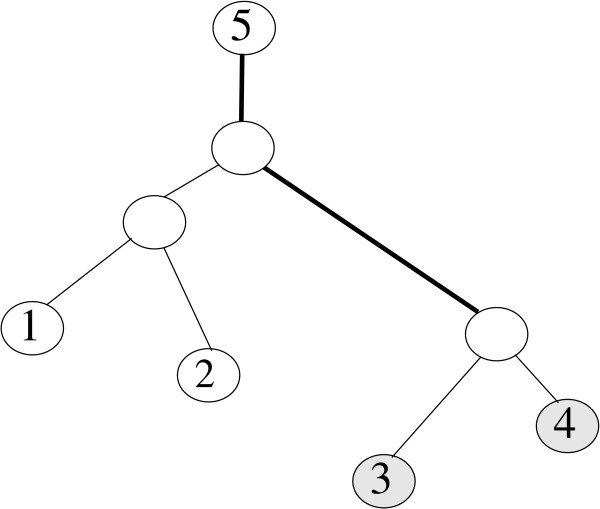
The tree is rooted at leaf 5. In the tree, leaves 3 and 4 with the largest path length from their most recent common ancestor to the root 5 are neighbors.

Our algorithm starts with the observation that if we take an arbitrarily rooted tree *T*_*m *_with *m *∈ *S *and length function , then there must be a pair of leaves (*neighboring leaves*) that share an immediate most recent common ancestor *mrca *which is farthest away from the root *m *with respect to . In Figure 5, the pair (3, 4) satisfies this condition, we say this pair fulfills the *largest path length criterion*. The largest path length criterion easily generalizes to arbitrarily rooted subtrees *T*_*i *_and *T*_*j *_of *T*_*m*_, where all descendants from the roots of *T*_*i *_and *T*_*j *_are in the vertex sets *V*_*i *_or *V*_*j*_, respectively.

Let  be the set of rooted subtrees of *T*_*m *_(each leaf *l *∈ *L*_*m *_is considered as a rooted subtree *T*_*l*_). Now consider two disjoint rooted subtrees *T*_*i *_and *T*_*j *_of *T*_*m *_where *i*, *j *∈ *V*_*m*_. Then the distance ℓ(*m*, *mrca*|*mS*_*i*_*S*_*j*_) from the *mrca *of *T*_*i *_and *T*_*j *_to *m *is computed according to Equation 4, where *S*_*i *_and *S*_*j *_are the leaf sets of *T*_*i *_and *T*_*j*_, respectively. Then we pick

(, ) = *argmax*{ℓ(*m*, *mrca*|*mS*_*i*_*S*_*j*_) | *T*_*i*_, *T*_*j *_∈ }     (6)

as a pair of neighbors (if we detect more than one pair, we randomly select one). By construction, (, ) fulfills the largest path length criterion.

If *D *is additive, ℓ(*m*, *mrca*|*mS*_*i*_*S*_*j*_) is exactly the path length from the *mrca *of (*T*_*i*_, *T*_*j*_) to *m*. In other words, the path length from the *mrca *of (, ) to *m *is large stand (, ) is a true neighboring pair. However, in real applications *D *is rarely additive, therefore the root *m *is selected so as to avoid noise from stochastic errors involved with large distance estimates [17]. To this end, *m *is selected such that the distance from the farthest object to root *m *is minimal,

*med *= *argmin*_*m*'∈*S*_{*max*{*D*(*m*'*x*)|*x *= 1,..., *n*}}     (7)

*med *is called *a median object*.

Moreover, to reduce the computational complexity of finding a pair of neighbors (, ) using Equation 6, we store for each *T*_*i *_∈  its *potential neighbor T*_*i*' _∈  such that

*T*_*i*' _= *argmax*{ℓ(*med*, *mrca*|*med*, *S*_*i*_, *S*_*j*_)|*T*_*j *_∈ }.     (8)

Now the neighboring pair (, ) fulfilling the largest path length criterion is determined as follows

(, ) = *argmax*{ℓ(*med*, *mrca*|*med*, *S*_*i*_, *S*_*i*'_)|*T*_*i *_∈ }.     (9)

In the following, we present a natural clustering algorithm to reconstruct trees based on distance matrices and the largest path length criterion

#### Clustering Algorithm

• **Initial step: **Find the median object *med *using Equation 7. Set  = {*T*_1_,..., *T*_*n*_} - {*T*_*med*_}. Find for each *T*_*i *_∈  its *potential neighbor T*_*i*' _∈  using Equation 8.

• **Selection step (largest path length criterion): **Find the neighboring pair (, ) using Equation 9.

• **Agglomeration step: **Combine  and  into a new rooted tree  with root *i*_0_*j*_0_, and estimate new edge lengths of  using Equation 5. Delete  and  and add  to . Find the potential neighbor for the new rooted tree . using Equation 8, and replace *T*_*i*' _for each *T*_*i *_∈  by  if  is its potential neighbor.

• **Stopping step: **If || > 1 goto the Selection step, otherwise output the tree.

This algorithm is similar to approaches described elsewhere [19-21], however, an essential difference is that we estimate path lengths and edge lengths by using triplets.

## Local rearrangement

The heart of the clustering algorithm is the largest path length criterion, at which the path length from the *mrca *of (*T*_*i*_, *T*_*j*_) to *med *is estimated by ℓ(*med*, *mrca*|*med*, *S*_*i*_, *S*_*j*_) using Equation 4. Thus, as path length we take the average of the lengths obtained from at most *O*(*n*^2 ^triplets {*med*, *s*_*i*_, *s*_*j*_} ∈ *med *× *S*_*i *_× *S*_*j*_. This average may not be the representative estimate of the true path length. Moreover the root *med *may be too far way from the *mrca *and this leads to an inaccurate estimate of the path length.

**Figure 6:**
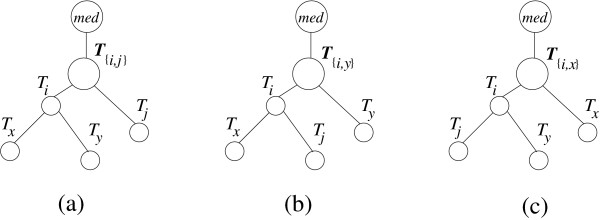
Reconstruction of new rooted tree *T*_{*ij*} _using the the preorder traversal procedure based on the largest average path length criterion. If (*T*_*x*_, *T*_*y*_) is the neighboring pair, we stick to the suggested grouping of *T*_*i *_and *T*_*j *_(see Figure 6a). Otherwise, if (*T*_*x*_, *T*_*j*_) or (*T*_*y*_, *T*_*j*_) is the neighboring pair, we switch to the trees displayed in Figure 6b or 6c, respectively.

To take these problems into account, we extend the clustering algorithm. To this end, imagine the algorithm has clustered *T*_*i *_and *T*_*j *_with corresponding disjoint leaf sets *S*_*i*_, *S*_*j *_⊂ *S *(we have finished the agglomeration step). Thus, we have created the newly rooted tree *T*_{*ij*} _with leaf set *S*_*ij *_= {*S*_*i *_∪ *S*_*j*_} and the set of unclassified objects *S*_0_(*S*_*ij*_) = *S *- *S*_*ij*_. In the following, we describe the nearest neighbor interchange operation around the root of *T*_*i *_upon condition that *T*_*i *_consists of two rooted subtrees *T*_*x*_, *T*_*y *_(Figure 6a). First, we estimate average path lengths from the unclassified object set *S*_0_(*S*_*ij*_) to the *mrca *of (*T*_*x*_, *T*_*y*_), (*T*_*x*_, *T*_*j*_) and (*T*_*y*_, *T*_*j*_) as







For convenience, we will use ℓ(*S*_0_(*S*_*ij*_)|*S*_*x*_*S*_*y*_) instead of ℓ(*S*_0_(*S*_*ij*_)*S*_*x*_*S*_*y*_|*S*_*x*_*S*_*y*_). We now use the average path lengths from Equation 10 to decide which pair of subtrees among (*T*_*x*_, *T*_*y*_), (*T*_*x*_, *T*_*j*_) and (*T*_*y*_, *T*_*j*_) is preferred. More specifically, if

ℓ(*S*_0_(*S*_*ij*_)|*S*_*x*_*S*_*y*_) ≥ *max*{ℓ(*S*_0_(*S*_*ij*_)|*S*_*x*_*S*_*j*_), ℓ(*S*_0_(*S*_*ij*_)|*S*_*y*_*S*_*j*_)}

we stick to the suggested grouping of *T*_*x *_and *T*_*y *_(see Figure 6a). Otherwise, if ℓ(*S*_0_(*S*_*ij*_)|*S*_*x*_*S*_*j*_) or ℓ(*S*_0_(*S*_*ij*_)|*S*_*y*_*S*_*j*_) is larger than the remaining average path lengths, we swap *T*_*y *_and *T*_*j *_or *T*_*x *_and *T*_*j *_as displayed in Figure 6b or 6c, respectively. Note that, this decision can be considered as a correction of the largest path length criterion by taking all possible triplets into account. We call the correction the *largest average path length criterion*.

We now explain the preorder traversal procedure [31] to reconstruct the rooted tree *T*_*i *_using the nearest neighbor interchange operation based on the largest average path length criterion (*T*_*i *_is a subtree of *T*_{*ij*} _= (*T*_*i*_, *T*_*j*_)):

### Preorder traversal procedure (*T*_*i*_)

• **Step 1: **If *T*_*i *_is a single leaf, return.

• **Step 2: **Otherwise, *T*_*i *_consists of two subtrees *T*_*x *_and *T*_*y*_. Do the nearest neighbor interchange operation around the root of *T*_*i *_based on the largest average path length criterion (Equation 10). If *T*_*x *_and *T*_*j *_(or *T*_*y *_and *T*_*j*_) were exchanged, estimate new edge lengths using Equation 5.

• **Step 3: **Apply the preorder traversal procedure to two rooted subtrees of *T*_*i*_.

#### Representative sets and shortest triplets

For a set *S *of sequences (or taxa), the (genetic) distance matrix *D *is typically not additive due to stochastic errors [17]. Larger distances between two sequences are less accurately estimated. This leads to a low performance of both the clustering algorithm and the preorder traversal procedure for divergent data sets.

**Figure 7:**
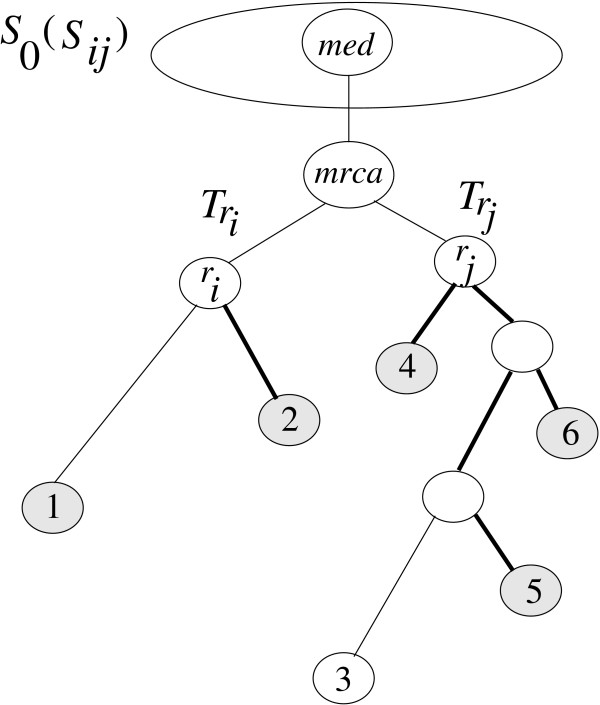
we select only *min*(*k*, |*S*_*i*_|) and *min*(*k*, |*S*_*j*_|) closest leaves to the root of *T*_*i *_and *T*_*j *_with respect to the path length, respectively, i.e. for *k *= 3 we pick {1, 2} from 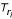 and {4, 5, 6} from 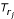. The leaf set {1, 2} (or {4, 5, 6}) is the 3-representative leaf set of the rooted subtree 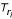. (or 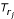).

In earlier work [10,11], we have presented simple representative concepts to reduce stochastic error involved in large distances. Here, we extend our work by introducing the so-called *k-representative set *concept. We use now genetic distances instead of topological distances (all edges have length 1). Our motivation is to reduce the computational complexity and to exclude objects far away from the root under consideration. In the clustering algorithm, the path length from the *mrca *of (*T*_*i*_, *T*_*j*_) to *med *(see Figure 7) can be estimated by two approaches. The first method picks randomly one pair (*s*_*i*_, *s*_*j*_) ∈ *S*_*i *_× *S*_*j *_then computes



The second approach takes the average distance



Both approaches suffer from noise. Estimating the path length using Equation 11 may be inaccurate because it randomly picks a pair (*s*_*i*_, *s*_*j*_) which may not be really representative. Equation 12 may be problematic, especially since it might be susceptible to noise, due to the possibility of including long- distances with large stochastic errors.

To overcome these problems, we select only *min*(*k*, |*S*_*i*_|) and *min*(*k*, |*S*_*j*_|) closest leaves to the root of *T*_*i *_and *T*_*j *_with respect to the path length, respectively. To illustrate, for *k *= 3 we pick {1, 2} from *T*_*i *_and {4, 5, 6} from *T*_*j *_in Figure 7.

We now define  as the set of *min*(*k*, |*S*_*i*_|) closest leaves to the root of *T*_*i*_.  is called the *k-representative leaf set*. Hereafter, we estimate similar to Equation 4 the path length from the *mrca *of (*T*_*i*_, *T*_*j*_) to *med *as



which is only based on the *k*-representative leaf sets. Now we can perform the clustering algorithm with reduced complexity. However, we also want to improve the preorder traversal procedure. The average path length from the unclassified object set *S*_0_(*S*_*ij*_) to the *mrca *of (*T*_*i*_, *T*_*j*_) is estimated by Equation 10 which also suffers from noise. To overcome this problem, we select only *min*(*k*, |*S*_0_(*S*_*ij*_)|) unclassified objects closest to the root of tree *T*_{*ij*} _with respect to distances  where *s*_0 _∈ *S*_0_(*S*_*ij*_). We call the subset, denoted (*S*_*ij*_), *k-representative unclassified object set*.

We now define  a *shortest triplet*, which contains three representatives of the three *k*-representative sets. By construction,  are close to the root of *T*_{*ij*} _and close to each other. Therefore, the edge length estimates based on shortest triplet {} are less susceptible to estimation errors.

We now rewrite Equation 10 to estimate the average path length from the representative unclassified object set (*S*_*ij*_) to the *mrca *of (*T*_*i*_, *T*_*j*_) using only shortest triplets as







In short, the preorder traversal procedure uses only shortest triplets to estimate path lengths as well as edge lengths.

#### Shortest triplet clustering algorithm (STC)

We introduce now the shortest triplet clustering algorithm by combining the clustering algorithm, the local rearrangement, the *k*-representative sets, and the shortest triplets approach.

### Shortest triplet clustering algorithm (STC)

• **Initial step:**

- **(i): **Find the median object *med *using Equation 7.

- **(ii): **Set  = {*T*_1_,..., *T*_*n*_} - {*T*_*med*_} and for each *T*_*i *_∈  its representative leaf set  = {*i*}.

- **(iii): **Find for each *T*_*i *_∈  its *potential neighbor T*_*i*' _∈  using Equation 8.

• **Selection step (largest path length criterion): **Find the neighboring pair (, ) using Equation 9.

• **Agglomeration step:**

- **(i): **Combine  and  into a new rooted tree  with root *i*_0_*j*_0_, and estimate new edge lengths of  using Equation 5 based on shortest triplets.

- **(ii): **Compute the *k*-representative leaf set  of . based on *k-*representative leaf sets  and  of  and , respectively.

- **(iii): **Compute the *k*-representative unclassified object set  of .

- **(iv): **Delete  and  and add  to .

- **(v): **Find the potential neighbor for the new rooted tree  using Equation 8 based on representative sets, and replace *T*_*i*' _for each *T*_*i *_∈  by  if  is its potential neighbor.

• **Local rearrangement step: **Apply the preorder traversal procedure to the rooted subtrees  and  of the new rooted tree  based on only shortest triplets.

• **Stopping step: **If || > 1, goto Selection step, otherwise output the tree.

### The complexity of STC

Now we briefly describe the complexity of the STC. At the initial step, (i), (ii), and (iii) are done in *O*(*n*^2^), *O*(*n*) and *O*(*n*^2^) time, respectively. Thus, the complexity of the initial step is *O*(*n*^2^). The selection step is done in *O*(*n*). At the agglomeration step, (i), (ii), (iii), (iv), and (v) are done in *O*(*k*^3^), *O*(*k*), *O*(*nk*^2^), *O*(1), and *O*(*nk*^2^) time, respectively. Thus, the complexity of the agglomeration step is *O*(*nk*^2 ^+ *k*^3^). Finally, we are estimating the complexity of the preorder traversal procedure based on only shortest triplets. Step 1 is done in constant time. Step 2, the nearest neighbor interchange operation around the root of *T*_*i *_costs *O*(*k*^3^). Estimating new edge lengths is done in *O*(*k*^3^) time. Re-computing the *k*-representative leaf set  of *T*_*i *_based on *k*-representative leaf sets of its rooted subtrees *T*_*x *_and *T*_*y *_costs *O*(*k*) time. Finally, re-computing the *k*-representative unclassified object set (*S*_*i*_) of *T*_*i *_based on the *k*-representative leaf set  of *T*_*j *_and the *k*-representative unclassified object set (*S*_*ij*_) of *T*_{*ij*} _is done in *O*(*k*) time. Thus, the complexity of step 2 is *O*(*k*^3^). Step 3 is done in constant time. Step 1, step 2, and step 3 are repeated *O*(*n*) times so the complexity of the preorder traversal procedure is *O*(*nk*^3^).

In the STC algorithm, the selection step, the agglomeration step and the local rearrangement step are repeated (*n *- 2) times so the overall complexity of the STC algorithm is *O*(*n*^2^*k*^3^). Practically, we chose *k *= 5 as a good compromise between the accuracy and computational complexity for all data sets. That is, the practical complexity of the STC algorithm is only *O*(*n*^2^).

## Authors' contributions

Both authors participated in the design of the study and performed the analysis. LSV implemented the algorithms. Both authors wrote and approved the final manuscript.

## Figures and Tables

**Table 1:** The average Robinson and Foulds distance of 100 simulated data sets of 500 sequences each with sequence lengths 500, 1000 and 2000 nt (rbcl simulation). Methods are used without BNNI.

sequence length	NJ	BIONJ	Weighbor	HGT/FP	GME	BME	**STC**^*k *= 5^
500	.190	.188	.194	.512	.240	.184	**.177**
1000	.100	.098	.099	.409	.144	.096	**.088**
2000	.049	.048	.050	.313	.082	.046	**.040**

**Table 2:** The average Robinson and Foulds distance of 100 simulated data sets of 500 sequences each with sequence lengths 500, 1000 and 2000 nt (rbcl simulation). Methods are used with BNNI.

sequence length	NJ	BIONJ	Weighbor	HGT/FP	GME	BME	**STC**^*k *= 5^
500	.162	.162	.162	.166	.163	.163	**.162**
1000	.079	.079	.079	.079	.080	.079	**.079**
2000	.035	.035	.035	.036	.036	.035	**.035**

**Table 3:** The average Robinson and Foulds distance of 100 simulated data sets of 1000 taxa for each tree diameter 0.1, 0.5, 1.0 and 1.5 and with sequence length 1000 nt (large simulation). Methods are used without BNNI.

number sequences	NJ	BIONJ	HGT/FP	GME	BME	**STC**^*k *= 5^
1000 (0.1)	.146	.146	.378	.168	.143	**.139**
1000 (0.5)	.093	.089	.193	.126	.075	**.066**
1000 (1.0)	.094	.090	.188	.132	.074	**.062**
1000 (1.5)	.097	.091	.182	.138	.073	**.061**

**Table 4:** The average Robinson and Foulds distance of 100 data sets of 5000 taxa for each tree diameter 0.1, 0.5, 1.0 and 1.5 and with sequence length 1000 nt (large simulation). Methods are used without BNNI.

number sequences	NJ	BIONJ	HGT/FP	GME	BME	**STC**^*k *= 5^
5000 (0.1)	.178	.179	.442	.207	.173	**.170**
5000 (0.5)	.109	.105	.210	.156	.084	**.072**
5000 (1.0)	.107	.102	.192	.155	.073	**.064**
5000 (1.5)	.112	.106	.188	.164	.072	**.063**

**Table 5:** The average Robinson and Foulds distance of 100 simulated data sets of 1000 taxa for each tree diameter 0.1, 0.5, 1.0 and 1.5 and with sequence length 1000 nt (large simulation). Methods are used with BNNI.

number sequences	NJ	BIONJ	HGT/FP	GME	BME	**STC**^*k *= 5^
1000 (0.1)	.137	.137	.137	.137	.137	**.138**
1000 (0.5)	.061	.061	.061	.061	.061	**.061**
1000 (1.0)	.057	.057	.057	.057	.057	**.056**
1000 (1.5)	.055	.055	.055	.055	.055	**.055**

**Table 6:** The average Robinson and Foulds distance of 100 data sets of 5000 taxa for each tree diameter 0.1, 0.5, 1.0 and 1.5 and with sequence length 1000 nt (large simulation). Methods are used with BNNI.

number sequences	NJ	BIONJ	HGT/FP	GME	BME	**STC**^*k *= 5^
5000 (0.1)	.168	.168	.168	.168	.168	**.168**
5000 (0.5)	.066	.066	.066	.066	.066	**.066**
5000 (1.0)	.057	.057	.057	.057	.057	**.057**
5000 (1.5)	.055	.055	.055	.055	.055	**.055**

**Table 7:** The average RF distance of the 96-taxon alignments of sequence length 500 nt, that were analyzed in [6]. The 6000 trees were split into three groups called "slow" (0.2 substitutions per site), "moderate" (0.4 substitutions per site) and "fast" (1.0 substitutions per site). Except for STC, the accuracies for the other methods were taken from [6]. Methods are used without BNNI.

number sequences	NJ	BIONJ	Weighbor	HGT/FP	GME	BME	**STC**^*k *= 5^
96 (slow)	.183	.180	.178	.512	.199	.186	**.179**
96 (moderate)	.136	.134	.129	.480	.158	.137	**.125**
96 (fast)	.115	.112	.103	.465	.144	.117	**.102**

**Table 8:** The average RF distance of the 96-taxon alignments of sequence length 500 nt, that were analyzed in [6]. The 6000 trees were split into three groups called "slow" (0.2 substitutions per site), "moderate" (0.4 substitutions per site) and "fast" (1.0 substitutions per site). Except for STC, the accuracies for the other methods were taken from [6]. Methods are used with BNNI.

number sequences	NJ	BIONJ	Weighbor	HGT/FP	GME	BME	**STC**^*k *= 5^
96 (slow)	.173	.173	.173	.175	.173	.173	**.173**
96 (moderate)	.119	.118	.118	.123	.118	.118	**.116**
96 (fast)	.090	.090	.091	.098	.091	.090	**.090**

**Table 9:** The percentage of cases where STC is at least as good as other tested methods in terms of RF distance. The number in parentheses is the percentage of cases where STC is equally good as other tested methods. Methods are used without BNNI.

number sequences	NJ	BIONJ	Weighbor	HGT/FP	GME	BME
96 (500 nt)	68 (16)	65 (15)	57 (16)	100 (0)	73 (10)	70 (14)
500 (500 nt)	80 (4)	76 (4)	88 (3)	100 (0)	100 (0)	66 (1)
500 (1000 nt)	88 (3)	79 (4)	84 (4)	100 (0)	100 (0)	83 (6)
500 (2000 nt)	92 (6)	90 (4)	92 (3)	100 (0)	100 (0)	86 (9)
1000 (1000 nt)	95 (2)	95 (1)	n.d.	100 (0)	100 (0)	89 (15)
5000 (1000 nt)	100 (0)	99 (0)	n.d.	100 (0)	100 (0)	95 (1)

**Table 10:** The percentage of cases where STC is better than other tested methods in terms of RF distance. The number in parentheses is the percentage of cases where STC is worse than other tested methods. Methods are used with BNNI.

number sequences	NJ	BIONJ	Weighbor	HGT/FP	GME	BME
96 (500 nt)	9 (8)	8 (8)	10 (10)	12 (10)	10 (8)	10 (9)
500 (500 nt)	34 (37)	35 (39)	35 (36)	59 (29)	46 (33)	41 (39)
500 (1000 nt)	22 (19)	17 (23)	18 (22)	23 (28)	30 (20)	24 (20)
500 (2000 nt)	10 (13)	8 (7)	10 (8)	9 (8)	12 (10)	7 (10)
1000 (1000 nt)	30 (28)	27 (29)	n.d.	28 (22)	30 (24)	28 (27)
5000 (1000 nt)	48 (40)	42 (44)	n.d.	45 (45)	52 (37)	43 (43)
